# Increasing zinc concentration in maize grown under contrasting soil types in Malawi through agronomic biofortification: Trial protocol for a field experiment to detect small effect sizes

**DOI:** 10.1002/pld3.277

**Published:** 2020-10-22

**Authors:** Lester Botoman, Patson C. Nalivata, Joseph G. Chimungu, Moses W. Munthali, Elizabeth H. Bailey, E. Louise Ander, R. Murray Lark, Abdul‐Wahab Mossa, Scott D. Young, Martin R. Broadley

**Affiliations:** ^1^ Crop and Soil Sciences Department Lilongwe University of Agriculture and Natural Resources (LUANAR) Bunda Campus Lilongwe Malawi; ^2^ Department of Agricultural Research Services Chitedze Agricultural Research Station Lilongwe Malawi; ^3^ School of Biosciences University of Nottingham Loughborough UK; ^4^ Inorganic Geochemistry Centre for Environmental Geochemistry British Geological Survey Nottingham UK

**Keywords:** effect size, power analysis, soil type, zinc‐enriched fertilizers

## Abstract

The prevalence of micronutrient deficiencies including zinc (Zn) is widespread in Malawi, especially among poor and marginalized rural populations. This is due to low concentrations of Zn in most staple cereal crops and limited consumption of animal source foods. The Zn concentration of cereal grain can be increased through application of Zn‐enriched fertilizers; a process termed agronomic biofortification or agro‐fortification. This trial protocol describes a field experiment which aims to assess the potential of agronomic biofortification to improve the grain Zn concentration of maize, the predominant staple crop of Malawi. The hypotheses of the study are that application of Zn‐enriched fertilizers will create a relatively small increase in the concentration of Zn in maize grains that will be sufficient to benefit dietary supplies of Zn, and that the effectiveness of agronomic biofortification will differ between soil types. The study will be conducted at three sites, Chitedze, Chitala, and Ngabu Agricultural Research Stations, in Lilongwe, Salima, and Chikwawa Districts respectively. These three sites represent locations in the Central and Southern Regions of Malawi. At each site, two different sub‐sites will be used, each corresponding to one of two agriculturally important soil types of Malawi, Lixisols, and Vertisols. Within each sub‐site, three Zn fertilizer rates (1, 30, and 90 kg/ha) will be applied to experimental plots using standard soil application methods, in a randomized complete block design. The number of replicates at plot level has been informed by a power analysis from pilot study data, assuming that a minimum 10% increase in Zn concentration of grain at 90 kg/ha relative to the concentration at 1 kg/ha is of interest. Grain mass (yield), stover mass, and both stover and grain Zn concentrations will be measured at harvest. A second year of cropping will be used to establish whether there are any residual benefits to grain Zn concentration. The potential for Zn agronomic biofortification will be communicated to relevant academic and government stakeholders through a peer review journal article and a briefing paper.

## 1 INTRODUCTION

Zinc (Zn) is an essential micronutrient, having critical physiological and biochemical functions in biological systems (Broadley et al., 2007). A lack of dietary Zn in humans can cause Zn deficiency with wide ranging effects on health (Brown et al., 2001). Often, Zn deficiency in humans is attributed to low dietary diversity and high levels of phytate consumption which inhibits Zn uptake in the human gut (Gibson & Hotz, 2001), in combination with consumption of food crops produced on Zn‐deficient soils (Gregory et al., 2017; Miller & Welch, 2013; Noulas et al., 2018). Dietary deficiencies of Zn and other micronutrients, especially in Low‐ and Middle‐Income Countries (LMICs), are reported to cause great economic losses and have a considerable effect on the gross national product (GNP) by decreasing productivity and increasing health care costs (Stein, 2014).

Malawi (population 17.5 million people; NSO, 2019) is one of the countries in sub‐Saharan Africa (SSA) with a high prevalence of Zn and other micronutrient deficiencies (Hurst et al., 2013; Manzeke et al., 2016; Phiri et al., 2019, 2020; Siyame et al., 2013). For example, Zn deficiency is reported to be widespread with a prevalence rate of 62% based on serum Zn concentration (Likoswe et al., 2020; NSO, 2017), with higher prevalence rates observed in rural populations (Siyame et al., 2013). Malawi has a small average Zn dietary supply of less than 14 mg capita^−1^ day^−1^ as compared with a supply of approximately 20 mg capita^−1^ day^−1^ in higher‐income countries such as the UK (Kumssa et al., 2015). This small dietary Zn intake is attributed to the importance of maize, which dominates the diet of most Malawians. Maize grain has inherently low Zn concentrations and high phytate concentrations, which inhibits the absorption of Zn and other minerals in the human gut (Joy et al., 2015, 2017;  Manary et al., 2002). Furthermore, many of Malawi's soils are typically highly weathered and contain insufficient plant‐available Zn for optimal crop growth and this can also lead to lower grain Zn concentrations. For example, in a survey of maize grain Zn concentration in Malawi, the concentration in grain grown on Vertisols was 30% larger than the grain concentration from other soils (Chilimba et al., 2011). Among subsistence smallholder communities farming on these Vertisols, there was a 35% greater dietary Zn supply than from other soils in Malawi, based on the analyses of composite diets (Siyame et al., 2013) and household‐level dietary recall (Joy, et al., 2015).

Possible solutions to human Zn deficiency via food systems interventions include dietary diversification, food fortification, and biofortification, which can encompass plant breeding and agronomic biofortification through application of Zn‐enriched fertilizers (Bouis et al., 2011; Cakmak, 2008; Wang et al., 2016). In subsistence contexts where consumption of processed foods is small, agronomic biofortification of staple food either through foliar application or through the soil is a potentially cost‐effective option. Joy et al. (2015) reviewed the potential of Zn‐enriched fertilizers to alleviate human dietary Zn deficiency and reported that an 18% increase in maize grain Zn concentration can be achieved by soil application of Zn‐enriched fertilizers. However, more studies across varied environmental conditions are needed to verify the estimated effects of applied Zn on maize grain concentration.

The use of Zn‐enriched fertilizers to increase maize grain Zn concentration can be improved when integrated soil fertility management (ISFM) practices are used, such as application of organic manures in combination with and maize‐legume intercropping systems. For example, in Zimbabwe, Manzeke et al. (2014) reported substantial increases in maize grain Zn concentrations when soil Zn fertilizers were used in combination with organic and mineral fertilizers. Thus, grain Zn concentration increased from a baseline of 13.5 mg/kg to 28.0 and 32.4 mg/kg, when either cattle manure or woodland leaf litter, respectively, was used in combination with soil Zn fertilizers. Even in the absence of soil Zn fertilizers, small increases in grain Zn concentration have been noted in maize when mineral NPK and organic fertilizers are used, in both experimental (Manzeke et al., 2012) and farmer surveys (Manzeke et al., 2019). Manzeke et al. (2020) reported increases in maize grain Zn concentration when nitrogenous fertilizers were co‐applied with foliar Zn fertilizer; with a 40% larger grain Zn concentration than when Zn was applied without nitrogen fertilizers. In Nigeria and Togo, Kihara et al. (2020) observed that application of Zn and other macronutrients caused an increase of about 20% in maize grain Zn concentration over a control treatment. In Pakistan, with seed priming, Harris et al. (2007) reported that maize grain Zn concentration was significantly higher (18.3 mg/kg) in a crop grown from seeds primed with a dressing containing 1% Zn than in a non‐primed crop (15.4 mg/kg).

Agronomic biofortification with Zn is not yet promoted in Malawi, although the Ministry of Agriculture and Food Security (MoAFS) has mandated the incorporation of 1% elemental Zn in granular basal fertilizers since 2016 to potentially improve crop yields (MoAFS, 2016). Although foliar application of Zn is likely to be more effective than soil application at increasing grain Zn concentration, due to the capacity of soils to fix Zn in unavailable forms, the method would be difficult to deploy by smallholder farmers especially over larger areas, due to the lack of access to machinery for spraying crops (Joy, et al., 2015). Furthermore, unlike wheat which is short‐statured and likely to be better at translocating Zn from leaf to grain (Doolette et al., 2018), foliar application is more difficult with the taller maize crop.

The current experiment aims to assess the potential of agronomic biofortification by soil application of Zn‐enriched fertilizers to improve Zn concentration in the edible part of maize in Malawi. The primary objective of the study is to determine the extent to which the application of Zn‐enriched fertilizers to soils will increase the concentration of Zn in grains. This effect might be in the region of 10%–20% (Joy, et al., 2015; Manzeke et al., 2012, 2017). The design of the experiment was therefore informed by a power analysis to detect a 10% effect. This is a novel approach to agronomic biofortification studies; typically, studies on maize (and other crops) have limited replication and therefore limited power to detect small effect sizes (Button et al., 2013; Lark et al., 2020; Uttley, 2019). A second objective is to determine how the effectiveness of agronomic biofortification will differ between Lixisols and Vertisols, with maize grown on Vertisols expected to have higher baseline grain Zn concentrations (Chilimba et al., 2011). Fertilizer use efficiency is affected by application methods. For example, Zhang et al. (2013) reported that the uptake of Zn by maize was influenced by where the fertilizer was placed in the soil, which affected the supply capacity of Zn in the soil. The current trial focuses on soil applications using the manual ‘spot’ (or ‘dollop’) application method, which is the standard agronomic approach of Malawian smallholder farmers according to national recommendations (MoAFS, 2018). For the spot method, a hole with a diameter of 2 cm and a depth of 10 cm is typically made 12.5 cm away from the maize planting station, using a stick, and fertilizers are then placed in the hole. To ensure uniform distribution of fertilizer, small cups of different sizes are calibrated to achieve target application rates. The applied fertilizers in the holes are then covered with soil. Given the large Zn application rate planned, it is also useful to ascertain whether there might be a residual benefit in terms of availability of soil Zn to subsequent crops. The experiment is therefore planned to run over two years; in the second year, maize will be grown on the same plots and ridges, without any added Zn. No further ploughing or ridging will be conducted.

## 2 MATERIALS AND METHODS

### 2.1 Materials

Maize (*Zea mays* L.) was chosen as the test crop because it is a staple food crop for Malawians. The F_1_ hybrid variety SC 403 was chosen because it is widely grown in Malawi, is early‐maturing, tolerates a wide range of environmental conditions, can mature in ~90 days, and has a high yield potential of up to 10,000 kg/ha (Seedco Malawi Ltd).

### 2.2 Pilot study

A pilot study was conducted at Chitedze, Chitala and Bembeke Agricultural Research Stations, in Lilongwe, Salima and Dedza Districts respectively, all in the Central Region of Malawi during the 2018–2019 cropping season. The soil types under study were Lixisols (Chitedze and Chitala) and Ferralsols (Bembeke), from the World Reference Base (WRB) of the IUSS Working Group WRB (2006). The aim of the pilot study was to conduct a preliminary assessment of the effectiveness of agronomic biofortification through application of Zn‐enriched fertilizers using both soil (spot application) and foliar application methods in increasing Zn grain concentration. The test crop was maize (variety SC 403). The study was laid out in a randomized complete block design (RCBD) with all the treatments replicated three times at each site. The gross plot size (including guard rows) was five ridges, each of 5 m length, with the net plot used for experimental measurements being the three middle ridges, each of 3 m length. The ridges were spaced at 75 cm distance. Plants were sown at 25 cm spacing along the ridge, giving an expected plant density of 53,333 plants ha^−1^. The pilot study used the following Zn fertilizer rates: 0, 1, 5, 10, and 20 kg/ha of elemental Zn, applied as a commercial grade ZnSO_4_.7H_2_O (22% elemental Zn; M.R. Zinc, Dalview, South Africa) in three equal splits at 1 week after planting, 3 weeks after planting, and at tasseling. The foliar application was made using a knapsack sprayer at a water application rate of 666 L/ha using a single pass for each plot with different concentrations corresponding to the respective Zn application rates in three equal splits at 1 week after planting, 3 weeks after planting, and at tasseling. Good agronomic practices were followed according to recommended guidelines as outlined in the Guide to Agricultural Production and Natural Resource Management (MoAFS, 2018). After harvesting, grain mass (yield), stover mass, and both stover and grain Zn concentrations were measured using methods described in Section 2.7. Grain Zn concentration data were used to support the power calculations for the main experiment, as described in Section 2.5.

### 2.3 Description of the experimental sites

The study will be conducted at Chitedze, Chitala and Ngabu Agricultural Research Stations in Lilongwe, Salima and Chikwawa Districts, respectively, during the 2019–2020 cropping season. Lilongwe and Salima are in the Central Region, and Chikwawa is in the Southern Region of Malawi. Table 1 describes the location of the experimental sites. All three sites are characterized by a unimodal rainfall pattern that normally comes from November to April. In Malawi, maize is typically sown in November, at the onset of the rainy season, and harvested in May or June, starting earlier in the south and low‐lying areas. The three sites are generally expected to experience a cool, dry period from May to mid‐August, with temperatures ranging from 16 to 27°C, then warmer from mid‐August to October with temperatures ranging from 20 to 40°C. From November to April, the sites are warm and wet with a temperature range of 25 to 36°C.

**Table 1:** Description of the experimental sites

Location	Selected soil type^a^	Geo‐reference	Elevation (masl)	Agro‐ecology
Chitedze	Lixisol	13.99 S, 33.64 E	1,150 m	Mid‐altitude plateau
	Vertisol	13.98 S, 33.65 E		
Chitala	Lixisol	13.69 S, 34.25 E	600 m	Lakeshore plain
	Vertisol	13.68 S, 34.26 E		
Ngabu	Lixisol	16.50 S, 34.86 E	100 m	Lower Shire valley
	Vertisol	16.45 S, 34.89 E		

Abbreviation: masl, meters above sea level.

^a^ Soil type based on World Reference Base (WRB) soil classification.

At each site, two sub‐sites have been selected so that the contrast between Lixisol and Vertisol soil types can be studied. These soil classes are from the World Reference Base (WRB) of IUSS Working Group WRB (2006) as applied in Malawi by Dijkshoorn et al. (2016). Vertisols are mostly dominated by 2:1 alumino‐silicate clay minerals and have a pH of greater than 7 and variable organic matter content (Kanwar, 1982). Because of the properties of 2:1 clay minerals, Vertisols undergo considerable shrinkage and swelling on drying and rewetting which results in large deep cracks in dry conditions which close only after prolonged wetting. In contrast, Lixisols have a pH range of 5.6–6.7 with a moderately high abundance of basic cations (Ca, Mg, K, and Na), a low available water holding capacity, and low‐activity clays at the depth of up to 100 cm (IUSS Working Group WRB, 2006).

These soil types were selected because of their significance for staple food crop production in Malawi and because there is evidence that Zn grain quality differs between maize crops grown on these two soils (Chilimba et al., 2011). Vertisols are naturally more fertile and productive than Lixisols and they are an agriculturally important soil type in Malawi despite occupying only a small proportion (1.5%) of the land area (Dijkshoorn et al., 2016; Ligowe, Young, et al., 2020). For example, they cover almost all (99%) of the area used by smallholder farmers for wetland‐irrigated farming (known locally as *dambo* farming) to produce green maize and vegetables (MoAFS, 2019). Smallholder wetland‐irrigated farming covers ~62,000 ha with an average maize yield of 1.1 t/ha to produce ~70,000 tonnes of maize per year. Of this total, 70% (i.e. ~49,000 tonnes) is consumed, boiled or roasted as green maize (MoAFS, 2019). Lixisols occupy 19% of land surface in Malawi (Dijkshoorn et al., 2016) and so play a significant role in crop production, although they are less productive than Vertisols.

The experimental areas were selected as having homogeneous soils. Prior to starting the experiment, soil samples will be collected from five points randomly spaced across the whole experimental area of each soil type at each site, at a depth of 0–20 cm. The collected soils will be thoroughly mixed, and a 500 g composite sample taken and analyzed for baseline soil characteristics, including plant‐available soil Zn status (Section 2.8).

### 2.4 Zn fertilizer treatments

Soil Zn applications are the focus of the study given that foliar Zn fertilizers are unlikely to be a realistic option in logistical terms for smallholder farmers in Malawi. A commercial grade Zn fertilizer will be used (ZnSO_4_.7H_2_O; 22% elemental Zn; M.R. Zinc, Dalview, South Africa). The trial will receive the following treatments; 1, 30, and 90 kg/ha with basal application at maize growth stage of three leaves, applied to plots on the contrasting soils at the sub‐sites. In terms of application method, all the rates will be applied as a basal application, when the crop is at the three‐leaf stage, by manually placing the fertilizers using the ‘spot’ (or ‘dollop’) method at 10 cm depth and 12.5 cm away from the planting station at a right angle to the ridge axis. The choice of the 30 and 90 kg/ha treatments in the main experiment is based primarily on observed low Zn grain concentration in the pilot trial, where a lower maximum rate of Zn application of 20 kg/ha was used, as described in Section 2.2. A zero Zn application rate was omitted in the current experiment and 1 kg/ha was adopted, because it is the content of the basal commercial fertilizers currently available in Malawi.

### 2.5 Experimental design, power assessment, and statistical analysis

At each of the six sub‐sites (one on a Lixisol and one on a Vertisol at each of the three sites), the three Zn fertilizer treatments will be applied to the three plots allocated within 10 complete randomized blocks, for which the experimental power can be explored. The choice of number of blocks is critical to determine the sensitivity of the experiment to detect responses to the fertilizer. For this reason, we undertook a power analysis. Because of the complexity of the experimental design, with blocking at the sub‐site level and fertilizer rates randomized in these blocks, we did this by simulation. A nested analysis of variance for the data on grain Zn concentration in the pilot trial provided an estimate of the variance components for the random variation around treatment responses for the between‐plot within‐block effects, and the between‐block within site effects. With just three sites, we did not examine the between‐site variance component, but we approximated this from the correlated variance component of the variogram for grain Zn concentration from a national survey of maize grain carried out in Malawi as part of the GeoNutrition project (Gashu et al., 2020; Ligowe, Phiri, et al., 2020). This variance will be somewhat inflated by effects of variation between soil types at national scale, so it is conservative to treat it as an estimate of the between‐site variance component in our proposed experiment. The between‐sub‐site variance component was set at the difference between the nugget variance for the national‐scale variogram and the sum of the between‐plot and between‐block variances from the pilot trial. The variance component values used for the power simulations are presented in Table 2.

**Table 2:** The variance component values of the pilot trial

Component and source	Variance (mg^2^ kg^−2^)
Between‐plot within‐block (pilot trial)	13
Between‐block (pilot trial)	0.1
Between‐soil sub‐site within site	2
Between site	3

For a proposed design, with some number of replicates (blocks), *B*, within each soil sub‐site a covariance matrix could be computed using the design matrices and the variance components above, for the random effects in the model. The function rmvnorm from the MASS package for the R platform (Venables & Ripley, 2002) was then used to generate a realization of random normal experimental errors with the specified covariance matrix. The fixed effects in the simulated data were specified. Mean Zn concentrations in grain for the Lixisol and Vertisol at the minimal rate of Zn fertilizer (1 kg/ha) were specified from the study of Joy, et al. (2015), as the values they returned for soils ‘other than Vertisols’ (17 mg/kg dry weight) and Vertisols (22 mg/kg dry weight, both values rounded to the nearest mg). It was then assumed that a 10% increase in Zn concentration was achieved by applying the maximum Zn fertilizer rate (90 kg/ha), and a 3.33% increase from the intermediate rate (30 kg/ha). These are the effect sizes of interest. Note that this power analysis is for a single season. No attempt was made to estimate power for an experiment with repeated measurements on a second season because there was no basis for specifying the correlation of within‐plot random effects in successive seasons.

A data set simulated with the above fixed and random effects was then analyzed using the nlme package in R. The *P*‐value for the null hypothesis of no difference in mean Zn concentration among plots receiving the contrasting Zn rates was extracted. For some specified value of *B* this process was repeated 1,000 times. The proportion of realizations for which *p* < .05 was computed. This is an estimate of the power of the experiment, with *B* blocks at each subsite, to reject the null hypothesis at this level of evidence, given the underlying specified treatment effects. The confidence interval for this estimate was computed using the method of Blaker (2000) as implemented in the blakerci function of the PropCIs package for the R platform (Scherer, 2018).

**FIGURE 1:**
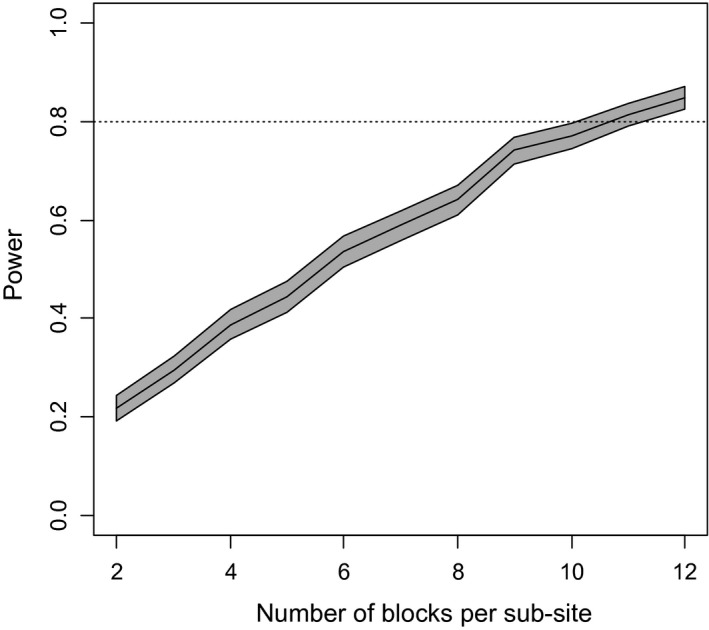
Power to detect a 10% effect size of fertilizer treatment on grain Zn concentration. The gray band shows the 95% confidence interval for estimated power to detect the specified fertilizer effects for differing numbers of blocks per sub‐site. The central line is the estimated power

Figure 1 below shows the plot of estimated power, and its 95% confidence interval, against *B* for values of the latter from 2 to 12. Preliminary discussion suggested that 10 blocks at each sub‐site would be a feasible rate of replication, given the logistical costs of field management and the amount of material for subsequent laboratory analysis. The plot shows that the estimated power at 10 blocks is 0.77. Given the inevitable uncertainties in all components of a power analysis based on assumptions about variance components, and the conventional target power of 0.80, it was decided that 10 replicates per sub‐site should be sufficient.

**FIGURE 2:**
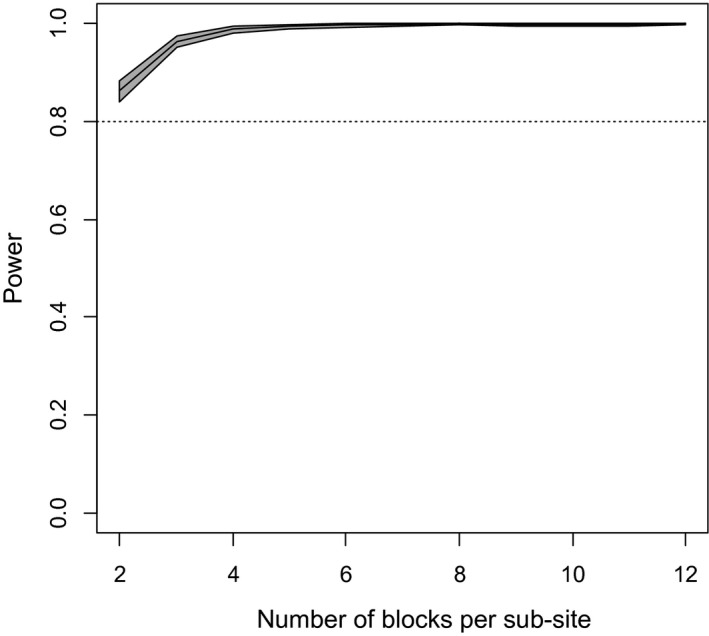
Power to detect a difference of 5 mg/kg between Lixisols and Vertisols. The gray band shows the 95% confidence interval for estimated power to detect the specified soil type effects for differing numbers of blocks per sub‐site. The central line is the estimated power

It was also possible to estimate from these same simulations the power of the experiment to detect the proposed soil type effect. The results are shown in Figure 2. It is apparent that the experiment is very adequately powered to examine soils effects.

**FIGURE 3:**
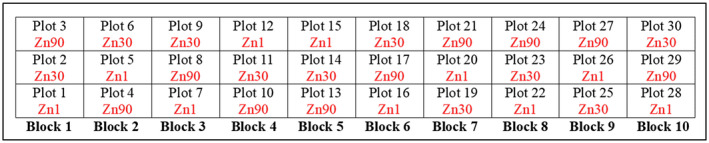
Plot layout on the Lixisol sub‐site at Chitedze Agricultural Research Station using independent randomization within each of 10 blocks. Key: Zn1 = 1 kg/ha, Zn30 = 30 kg/ha and Zn90 = 90 kg/ha

The allocation of treatments to plots within blocks was done independently and at random using a script for the R platform (R Core Team, 2017). Figure 3 shows the layout of plots in blocks for the Lixisol sub‐site at the Chitedze Agricultural Research Station.

Data analyses will be conducted using the nlme package for the R platform (Venables & Ripley, 2002). A linear mixed model will be used with a random effects structure to reflect how the fertilizer treatment is randomized among plots within sets of blocks all within one sub‐site of a single soil type. The random effects structure will also reflect dependence between the repeated measurements on the plots made in the two successive seasons of the experiment. We shall use a fixed effects model comprising main effects of fertilizer rate, soil type, and their interaction. We shall further partition the main effect of fertilizer rate into linear and non‐linear components with an appropriate choice of orthogonal polynomials, and similarly examine the partition of the soil‐fertilizer interaction into components based on these two components of the fertilizer effect. The output of the analysis will be tests of the specific hypotheses about differences between soil types and fertilizer application rates with respect to Zn concentration of grain and stover and estimates, with confidence intervals, of the effects of Zn fertilizer application on Zn concentration in grain at the 30 kg Zn ha^−1^ and 90 kg Zn ha^−1^ relative to the recommended rate of 1 kg Zn ha^−1^. Several studies indicate yield benefits with the application of Zn‐enriched fertilizers to maize crop (Cakmak, 2008; Kihara et al., 2020; Liu et al., 2016; Manzeke et al., 2012, 2014; de Valença et al., 2017). For example, Manzeke et al. (2014) reported a 29% increase in grain yield when Zn fertilizers was applied, which could offset the input costs of agronomic biofortification.

### 2.6 Trial establishment and management

The land will be ploughed and ridged with a tractor before the maize is planted as a monocrop. One seed will be sown per planting station, with planting stations spaced at 25 cm along the ridges of a gross plot comprising six ridges each of 5 m length, which are spaced at 75 cm. This represents an extra ridge per plot compared with the pilot study. The trials will be managed by the lead researcher (Lester Botoman) with support from technical officers from the Department of Agricultural Research Services (DARS) of the MoAFS. Technical officers will help with planting, weed management, fertilizer application and general trial management. All the treatments will receive adequate supply of nitrogen (N), phosphorus (P), potassium (K) and sulfur (S) based on Malawi's national fertilizer application recommendations (MoAFS, 2018). This will be achieved through basal application of calcium ammonium nitrate (27% N), single superphosphate (18% P_2_O_5_), and muriate of potash (60% K_2_O), all ‘Superfert’ brand (Liwonde, Malawi) at planting or soon after emergence and urea (46% N) as a top dressing at approximately 21 days after planting. None of these fertilizers contain Zn in their formula. A recommended application rate was adopted of 92 kg N/ha, 10 kg P_2_O_5_/ha, and 5 kg K_2_O/ha (MoAFS, 2018). In the second year, the study will examine the residual effects of applied Zn to determine its availability for uptake by maize crop. For this, the same planting and maintenance regime will be followed as described above, except Zn fertilizer will not be applied. In a study conducted to determine the residual availability of fertilizer Zn over a period of 5 years, Boawn (1974) observed substantial benefits of residual Zn in terms of uptake by maize even in 5th year. The study reported that Zn maize uptake was not significantly different throughout the whole 5‐year period. This suggests that applied fertilizer Zn remains available in the soil for subsequent growing seasons.

### 2.7 Sample and data collection and laboratory analysis

Plants from a net plot of the innermost four ridges, each of 3 m in length, will be harvested, and stover and grain samples prepared for analysis. Agronomic data will be collected from the net plots on the following: dry weight of grain (yield; kg), dry weight of stover (kg), and the seed weight of 100 kernels (kg) taken as a sub‐sample from the grain yield. Rainfall data (mm) will also be recorded, using rain gauges stationed in each of the research stations where the experiments will be conducted. For laboratory plant analysis, approximately 400 g of grain and a quantity of stover will be representatively sampled from each harvested net plot and dried in an oven at 105°C for 24 hr (Weinberg et al., 2008) to achieve a recommended moisture content of less than 14% (Likhayo et al., 2018; Weinberg et al., 2008). These representative samples will be obtained after the whole grain yield and biomass weight of the net plot is determined. The grain will be shelled and kernels will be thoroughly mixed in a bag and sample scooped from the bag. The reduction in moisture content will be verified using a grain moisture meter. After drying, stover samples will be chopped, and grain samples fine milled at Chitedze Agricultural Research Station, Malawi. A representative sub‐sample of ~50 g of each milled sample will be shipped for laboratory analysis at the School of Biosciences, University of Nottingham, UK in plastic write‐on panel bags. A portion of finely ground plant material (c. 0.2 g of grain or stover) will be digested with 6 ml of 70% HNO_3_ (trace analytical grade) using a microwave system comprising a Multiwave Pro 41HVT56 Rotor and pressure‐activated‐venting vessels made of modified polytetrafluoroethylene (PTFE‐TFM, 56 ml ‘SMART VENT’, Anton Paar GmbH, Graz, Austria). Two operational blanks will be included in each digestion run to enable the limit of detection (LOD) to be determined. Duplicate samples of a certified reference material (CRM: Wheat flour SRM 1567b, NIST, Gaithersburg, MD, USA) will be included in approximately every fourth digestion run to enable estimates of recovery to be determined. Following digestion, each tube will be made up to a final volume of 15 ml by adding 11 ml Milli‐Q water, then transferred to a 25 ml universal tube (Sarstedt Ltd., Nümbrecht, Germany) and stored at room temperature. Prior to analysis, samples will be diluted further 1:5 with Milli‐Q water into 13 ml tubes (Sarstedt Ltd.). Grain samples will be analysed for Zn by inductively coupled plasma mass spectrometry (ICP‐MS) (Mossa et al., 2020).

Zinc concentration in grains as a function of application rates and soil type will be determined and overall Zn uptake by the maize crops will be calculated as the summation of grain uptake and stover uptake (g/ha):

(1) $${\text{Zn}}_{\text{crop}}\left({\text{g ha}}^{‐1}\right)=Y\left({\text{kg ha}}^{‐1}\right)\times \frac{\left({\text{Zn}}_{x}\left({\text{mg kg}}^{‐1}\right)\right)}{1000}$$

where Zn_crop_ is the Zn content of the grain or stover, *Y* is the yield of grain or stover, and Zn*_x_* is the Zn concentration in the grain or stover:

(2) $${\text{Zn}}_{\text{uptake}}\left({\text{g ha}}^{‐1}\right)=\left({\text{Zn}}_{\text{grain}}+{\text{Zn}}_{\text{stover}}\right)$$

where Zn_uptake_ is the Zn maize uptake, Zn_grain_ is the Zn grain content, and Zn_stover_ is the Zn stover content.

To estimate the partitioning efficiency of Zn to grain, nutrient harvest index will be calculated to quantify the efficiency of maize in partitioning Zn to edible part of the crop:

(3) $${\text{Zn}}_{\text{HI}}=\left(\frac{{\text{Zn}}_{\text{grain}}\left({\text{g ha}}^{‐1}\right)}{{\text{Zn}}_{\text{uptake}}\left({\text{g ha}}^{‐1}\right)}\right)\times 100$$

where Zn_HI_ is the Zn Harvest Index, Zn_grain_ is the Zn grain content and Zn_uptake_ is the Zn maize uptake.

### 2.8 Measurements of residual availability of zinc in soil

The measurements of residual Zn in the soil prior to another crop being planted can determine the extent of its availability for the next crop. Such measurements might also be useful in assessing whether the ‘spot’ application location is optimal. The residual benefit of soil applied Zn to subsequent crops has previously been noted (Boawn, 1974; Brennan & Bolland, 2007; Grewal & Graham, 1999; Mari et al., 2015). After harvesting the trial, soil samples from the depth of 0–20 cm will be collected from all of the plots at Chitedze Research Station, from both the Lixisol and Vertisol sub‐sites. The soil sampling will be performed according to the methods described by Mari et al. (2015). Soil samples will be collected at ten points along the summit of one of the peripheral ridges, which will be selected at random from each net plot, using a Dutch soil auger with a flight length of 15 cm and a diameter of 3.5 cm, and the 10 samples from each plot will be bulked. To minimize potential Zn contamination, soil samples will be collected in the order of increasing amounts of Zn applied. The samples will be air‐dried, sieved (<2 mm) and homogenized before determination of extractable Zn as a measured of plant available Zn using the diethylenetriaminepentaacetic acid (DTPA) method (Lindsay & Norvell, 1978). The extraction will be undertaken in duplicates subsamples from each plot, using 5 g of soil, extracted with 10 ml 0.005 M DTPA, 0.1 M triethanolamine and 0.01 M CaCl_2_ at pH = 7.3 for 2 hr on an end‐over‐end shaker. Thereafter, the samples will be centrifuged at 1,900 ***g*** for 10 min and the supernatant filtered through <0.22 µm prior to analysis using ICP‐MS.

## 3 RESULTS AND DISCUSSION

The current trial is robust with an expected output to provide evidence on the effectiveness and efficiency of agronomic biofortification through spot application to soils of mineral Zn fertilizers in Malawi. In the short‐term, this strategy could be a cost‐effective way to alleviate Zn deficiency among the rural populations of developing countries such as Malawi. Increasing Zn to staple crops is reported to reduce people's nutritional vulnerability, because, when economic shocks occur, the consumption of higher‐value food commodities that are rich in Zn tends to reduce among lower‐income groups (Qaim et al., 2006). The results of the trial will help inform policy direction in agricultural sector in Malawi and other countries in addressing Zn micronutrient deficiency. It is also expected that results of the trial will be communicated to various stakeholders and reported through a peer review journal article.

## CONFLICT OF INTEREST

The authors declare no competing interests.

## AUTHOR CONTRIBUTIONS

LB conceptualized the study as part of his PhD studies, under the supervisory guidance of PCN (lead supervisor, LUANAR), JGC, MWM, EHB, ELA, RML, AWM, SDY, and MRB. LB led the pilot experimental work. RML led on statistical guidance. LB, PCN, RML, and MRB wrote the primary draft of the paper with editing and reviewing inputs from all other authors.

## Data Availability

Full results will be published in the Supplementary Tables of the paper reporting the experimental findings.
